# Metabolic acidosis after sodium thiosulfate infusion and the role of hydrogen sulfide

**DOI:** 10.1002/ccr3.1673

**Published:** 2018-07-01

**Authors:** Guenola M. Hunt, Hilary F. Ryder

**Affiliations:** ^1^ Walter Reed National Military Medical Center Bethesda MD USA; ^2^ Dartmouth‐Hitchcock Medical Center Lebanon NH USA; ^3^ Geisel School of Medicine at Dartmouth Hanover NH USA

**Keywords:** end‐stage renal disease, hydrogen sulfide, metabolic acidosis, sodium thiosulfate

## Abstract

Sodium thiosulfate (STS), first‐line treatment for calcific uremic arteriolopathy, causes a mild asymptomatic acidosis in many patients. However, severe, life‐threatening acidosis out of proportion with the expected acid load of STS may occur, potentially due to metabolism of STS to hydrogen sulfide.

## 1 INTRODUCTION

Calcific uremic arteriolopathy (CUA), or calciphylaxis, is an inflammatory condition in which calcium deposits on arteriolar vascular beds, leading to ischemia, cell damage, and sometimes necrosis. Sodium thiosulfate (STS) has emerged as a first‐line treatment for CUA ulcers and necrosis, but much remains unknown about its metabolism and toxicity. We present a case in which a patient developed severe metabolic acidosis with an anion gap following STS infusion, which was distinctly out of proportion with the expected metabolic changes. Hydrogen sulfide is a known metabolite of STS, and its role in hypoxia signaling in vascular beds and its neutralization of reactive oxygen species may contribute to the therapeutic role of STS. In chronic kidney disease (CKD), levels of hydrogen sulfide are reduced and restoring endogenous levels may be an important mechanism of STS action. The toxicity profile of hydrogen sulfide includes inhibition of mitochondrial ATP production and extreme lactic acidosis, possible contributor to the metabolic acidosis with an anion gap. Still, given the rarity of this reaction, some patients may possess a metabolic phenotype that confers a predisposition to this dangerous event.

Calcific uremic arteriolopathy is an inflammatory condition associated with end‐stage renal disease (ESRD) in which calcium salts deposit on arteriolar vascular beds, leading to ischemia, cell damage, and possibly necrosis.^1^ CUA is positively associated with female sex, obesity, diabetes, protein C deficiency, hyperphosphatemia, hypercalcemia, the use of calcium‐based phosphate binders, and warfarin use in patients requiring renal replacement therapy, but more cases are being seen with normal calcium and phosphate levels due to better standards of care.^1^ The incidence of CUA in ESRD has been documented for 5.7 cases per 10 000 chronic hemodialysis patients in 2011 and is rising^2^; prevalence as high as 4% has been documented 3 with variability attributed to differences to the years of the studies published and changing identification of the disease and management of risk factors contributing to CUA development. This is a serious condition, which can cause life‐threatening infections or organ failure when left untreated. Historically, CUA is associated with an overall poor prognosis and a mortality rate per year ranging between 64% and 80%. The mortality rate has likely decreased in the past decade due to treatment with sodium thiosulfate (STS), which has been adopted as standard of care without evidence derived from large trials.^1^ STS is regarded by many to be a benign treatment, with nausea, vomiting, and abdominal pain as the most frequent side effects.^1^ There is emerging evidence that a minority of patients may unpredictably experience metabolic acidosis with a very high anion gap, resulting in delirium, weakness, or cardiac arrhythmia.^4^ Here, we describe a metabolic acidosis that developed after STS infusion and review the literature to provide a potential explanation.

## 2 CASE REPORT

A 68‐year‐old Caucasian woman with end‐stage renal disease on dialysis was admitted to our facility for a stroke and a non‐ST segment elevation myocardial infarction. The stroke caused a right facial droop and right upper extremity flaccid paralysis, without any other sensory or cognitive disturbances. Her chronic medical conditions included type 2 diabetes mellitus (20‐year history, hemoglobin A1C 6.6% on admission), atherosclerotic coronary vascular disease, ischemic cardiomyopathy with an ejection fraction of 35%, congestive heart failure, paroxysmal atrial fibrillation on warfarin anticoagulation, hypertension, and hyperlipidemia. She had been receiving dialysis for 2 months and was anuric. Concurrently, she was found to have tender, nonhealing, necrotic, violaceous, ulcerating plaques of her right shin and left posterior calf, which were evaluated by dermatology and thought to be consistent with CUA. Biopsies were not obtained due to supratherapeutic INR and concern for inability to heal from biopsy. Antibiotics (clindamycin, later changed to vancomycin) were initiated. Blood pressure and rate control therapies with beta‐blockers were initiated to mitigate myocardial demand ischemia. She was beyond the time range for thrombolytic therapy, but her neurologic symptoms did not progress under observation.

The patient continued her scheduled intermittent hemodialysis (dialysate of 4 mEq/L potassium, 2.5 mEq/L calcium, and 35 mEq/L bicarb, duration of therapy 4 hours, ultrafiltration 3 kg) on the day of her admission after medical stabilization. During the last hour of the dialysis session that evening, 25 g of STS was also administered intravenously to treat her CUA ulcers. The morning following, the patient was found to have delirium, hypotension, and a metabolic acidosis with an elevated anion gap (Table 1). ABG revealed a primary metabolic acidosis with an increased anion gap, mixed with metabolic alkalosis with full respiratory compensation. Lactate and β‐hydroxybutyrate were not elevated. Sepsis was suspected; however, three sets of blood cultures were obtained and revealed no growth, and helical computed tomography scan of chest, abdomen, and pelvis revealed no nidus of infection. Her metabolic acidosis with anion gap did not resolve despite daily dialysis with dialysate of 35 mEq/L bicarb and stabilized with a supplemental bicarbonate drip; her anion gap trended downward over the next 4 days and eventually returned to her baseline.

**Table 1:** Relevant laboratory values during admission

	Admission (Hospital Day 0)	Post‐STS infusion (Hospital Day 1)	Hospital Day 2	Hospital Day 3	After Stabilization (Hospital Day 4)
pH Arterial	7.35	7.33			Not tested
pCO_2_ Arterial	41	31			Not tested
Lactate (mmol/L)	2.6	2.7			Not tested
Sodium (mEq/L)	134	139			135
Potassium (mEq/L)	4.7	4.8			4.5
Chloride (mEq/L)	91	88			90
HCO3 (mEq/L)	20	14			21
Anion Gap	23	38			20
Corrected Anion Gap	24.5	39.75			N/A
Blood Urea Nitrogen (mg/dL)	58	30			45
Creatinine (mg/dL)	5.41	3.98			3.20
Calcium (mg/dL)	9.1	9.7			7.2
Phosphorus (mg/dL)	3.9	5.5			5.0
β‐Hydroxybutyrate (mmol/L)	NA	2.31			1.84
Alkaline Phosphatase (mg/dL)	105	92			Not tested
Aspartate Transaminase (U/L)	41	33			Not tested
Alanine Transaminase (U/L)	13	12			Not tested
Albumin (gm/dL)	3.4	2.9			Not tested

After investigations for potential sepsis, ketosis, lactic acidosis, and uremia, STS was considered to be the probable instigating drug in her metabolic derangement. Due to the life‐threatening nature of this profound anion gap academia, the clinical team discontinued STS. She was treated conservatively with the help of our wound care team.

Without STS therapy, her ulcers continued to worsen. She was judged to not be a candidate for surgical debridement due to her multiple comorbidities. Her hospital stay was complicated by poor nutrition, health care‐associated pneumonia, and a *Clostridium difficile* infection. Thirty days into her hospital stay, she elected to switch to hospice care and died shortly thereafter.

## 3 DISCUSSION

Since its first described use in 2004 for CUA, STS been used more frequently, becoming the first‐line choice of treatment for this condition, which has been known in the community for its high morbidity.^1^, ^5^ Still, despite its longtime use in the medical and industrial domains, we understand relatively little about its pharmacodynamic effects in the body. In the literature, two types of acidosis produced by STS administration are described: one, a dose‐dependent, mild and asymptomatic acidosis present in most patients on STS therapy^6^ and another, a severe, life‐threatening acidosis out of proportion with the expected acid load of STS.^4^ Here, we discuss the known pharmacology of STS and discuss the properties of hydrogen sulfide, a metabolite of STS, that may play an additional role in both STS efficacy and side effects.^7^

In healthy patients, STS is excreted renally and hepatically in equal proportions.^8^ Previously, a component of biliary excretion based on canine models was reported, but that does not seem to be present in humans.^9^ A half‐life of 3 hours or more in healthy subjects has been cited previously,^9^ but newer investigations do not support this figure. 8 Total body clearance of STS occurs at a rate of 4.1 and 2.04 mL/min/kg in healthy subjects and chronic hemodialysis subjects, respectively.^8^ Based on these estimates, the higher plasma concentrations needed for optimal calcium chelation are sustained for about 25 minutes in subjects with ESRD, when STS is given after dialysis.^8^ The fast rate of plasma clearance implies that the effect of STS on chelating vascular calcification is perhaps less important than initially thought or that the by‐products of its metabolism are more therapeutically active than STS itself.

Supporting the role of metabolic by‐products are some in vitro studies with mouse models that explore the dynamics of calcium homeostasis in adipocytes and vascular smooth muscle cells.^10^ In high‐phosphate environments, as is seen with ESRD, adipocytes can start to express similar gene profiles to osteoclasts, leading to a phenotype change leading to cellular calcification.^10^ Further, adipocytes induce calcification of nearby vascular beds through upregulated VEG‐A. This effect in a high‐phosphorus environment can be dampened by the administration of STS, leading to a reduction in adipocyte phenotype changes and nearby vascular calcification for at least 3 days.^10^ This effect contrasts the found half‐life of STS itself, suggesting that a metabolite impacts transcription factor expression to mitigate calcification in the tissues.

In addition to formation of calcium thiosulfate via chelation and its effects on paracrine signaling, STS is thought to restore endothelial homeostasis by increasing nitric oxide synthase activity and regenerating glutathione.^1^ This multifactorial activity is perhaps responsible for the rapid response in pain control some patients experience, as their ability to vasodilate is restored before calcium homeostasis.^11^ The exact biochemical pathways of the reactions facilitated by STS remain uncharacterized, but they may be in part through the action of hydrogen sulfide, whose effects on glutathione, nitric oxide, and vasodilation pathways are already known.^7^ Endogenous thiosulfate has been hypothesized to act as a precursor for hydrogen sulfide to maintain homeostasis, and multiple enzymes in multiple tissue types facilitate the reaction from thiosulfate to hydrogen sulfide,^12^, ^13^ but the exact reaction under physiologic conditions remains uncharacterized.

Hydrogen sulfide is a key messenger in various pathways that coordinate the hypoxic vasodilation/vasoconstriction of vascular beds.^7^ It also can induce angiogenesis and independently protect cells from ischemia.^7^ In ESRD, endogenous levels of hydrogen sulfide are reduced, possibly affected by reactive oxygen species or uremia,^14^ but restoring hydrogen sulfide balance does not seem to be an active focus of patient care in ESRD.

In the case of severe, unexpected, and disproportionate metabolic acidosis, perhaps hydrogen sulfide can also be implicated. The toxicity of hydrogen sulfide was first discovered in accidental exposures of sewer workers to pockets of gas. With exposure, patients may experience mucosal irritation, pulmonary edema, loss of consciousness, and sometimes death.^15^ Mucosal irritation and pulmonary edema are seemingly related to direct gas exposures, and loss of consciousness and death are the results of excess sulfide in the blood.^15^ Excess hydrogen sulfide can inhibit oxidative phosphorylation, causing a shortage of ATP, ischemia, and profound lactic acidosis, an effect that can be quickly fatal when inhaled in high concentrations especially when combined with hypoxia.^7^, ^16^ When dissolved in the tissues in high concentrations, it inhibits the mitochondrial cytochrome complex IV in addition to directly causing profound sulfide‐generated oxidative stress. 16 This effect contrasts its ability to reduce oxidative stress in lower concentrations by downregulating pro‐ROS forming enzymes.^7^ While many people can receive 25 g of STS safely, perhaps there is a patient‐specific idiosyncratic reaction, such as a metabolic phenotype or a drug interaction, that we have not yet characterized. Further investigation into the pharmacokinetics of hydrogen sulfide and its behavior in ESRD is needed.

When thinking about our patient and the others with dangerous acidemia, it is still unknown why these events occur. With our current understanding of thiosulfate, hydrogen sulfide, and the physiology of ischemia, there are several possibilities: (1) the acid formed from thiosulfate becoming thiosulfuric acid in an aqueous solution during administration; (2) the direct hydrogen load of hydrogen sulfide; (3) lactic acid accumulation from ATP production inhibition; (4) an insurmountable oxidative stress from sulfide‐containing compounds; and (5) a rare phenotype that makes the above more likely to occur.

## 4 CONCLUSION

As STS use in ESRD populations increases, we are beginning to characterize side effects of STS, which include a mild acidosis from the administration of an acidic compound and a distinctly different and more extreme acidosis with an anion gap out of proportion with the acid load of the drug itself. We do not know why this reaction occurs in some patients or what the predisposing factors may be, but metabolites of STS may be responsible. Hydrogen sulfide is a ubiquitous inorganic compound in the body and has known effects on vascular inflammation and oxygen delivery. While still unexplored, the breakdown of thiosulfate to hydrogen sulfide may be responsible for the drug's efficacy. Similarly, the toxicity profile of hydrogen sulfide, as seen in accidental occupational exposures, may provide some clues as to why some patients develop profound metabolic acidosis. More research is needed into finding the cause of such a dangerous idiosyncratic reaction.

## CONFLICT OF INTEREST

None declared.

## AUTHORSHIP

All persons who meet authorship criteria are listed as authors, and all authors certify that they have participated sufficiently in the work to take public responsibility for the content, including participation in the concept, design, analysis, writing, or revision of the manuscript. GMH: collected data from the chart, analyzed the data, performed a literature search, and was the primary author of the manuscript. HR: reviewed the data collected from the chart, reviewed the literature, assisted with the writing of the manuscript, and performed a revision of the manuscript.
