# Supplementary material for: Metabolic acidosis after sodium thiosulfate infusion and the role of hydrogen sulfide
Source: Clin Case Rep. 2018 Jul 1;6(8):1595–9. doi: 10.1002/ccr3.1673 (PMC6099024; doi:10.1002/ccr3.1673)
Supplement: Supplementary file 1 [file CCR3-6-1595-s001.docx]

Sodium thiosulfate, first-line treatment for calcific uremic arteriolopathy, causes a mild asymptomatic acidosis in many patients. However, severe, life-threatening acidosis out of proportion to the expected acid load of STS may occur, potentially due to metabolism of STS to hydrogen sulfide.
